# Prognostic implication of monocytes in atrial fibrillation: The West Birmingham Atrial Fibrillation Project

**DOI:** 10.1371/journal.pone.0200373

**Published:** 2018-07-18

**Authors:** Farhan Shahid, Nur A. Rahmat, Gregory Y. H. Lip, Eduard Shantsila

**Affiliations:** Institute of Cardiovascular Sciences, University of Birmingham, Birmingham, United Kingdom; Osaka University Graduate School of Medicine, JAPAN

## Abstract

**Background and objectives:**

High monocyte counts are related to adverse outcomes in cardiovascular disease. Their role in prognostication in patients with atrial fibrillation (AF) is unknown. We investigated whether monocyte counts are useful as a marker of prognosis in patients with AF.

**Methods:**

Monocyte counts were obtained from blood samples in 881 AF patients. Study outcomes were (i) all-cause death; (ii) major adverse cardiovascular events; (iii) stroke, TIA or other systemic embolism (SSE); and (iv) major bleeding.

**Results:**

Median follow up was 7.2 years; 44% of patients died, 48% developed MACE; 9% had SSE and 5% had major bleeding. On Cox regression, after adjustment for CHA_2_DS_2_-VASc score, the highest quartile of monocyte counts (i.e., ≥580 μL vs. other quartiles) was associated with increased risk of death (hazard ratio [HR] 1.64, 95% confidence interval [CI] 1.31–2.05, p<0.001) and MACE (HR 1.58, 95% CI 1.28–1.96, p<0.001). Persistent monocyte levels ≥580 per μL during follow up were associated with further increase in risk of death (HR 1.52, 95% CI 1.10–2.11, p = 0.01) and MACE (HR 1.54, 95% CI 1.13–2.09, p = 0.006). Persistent monocyte levels ≥580 per μL during were associated with a significant increase in major bleeding events (HR 2.77, 95% CI 1.36–5.67, p = 0.005, after adjustment for HAS-BLED score).

**Conclusion:**

High monocyte counts independently predict the occurrence of MACE, major bleeding and mortality, but not SSE. Understanding the pathophysiological mechanisms involved would help understand the relationships between monocytes, and adverse thrombotic and bleeding outcomes in AF patients.

## Introduction

Circulating monocytes have been closely linked to outcomes in patients with cardiovascular disease[1]. The primary role of monocytes is to detect and replenish the stores of macrophages and dendritic cells, and to provide phagocytosis of pathogens[2]. Monocytes make up to 8% of the peripheral blood white cells and play a central role in the host response to infective agents, such as bacteria and viruses. Additionally, monocytes modulate the inflammatory processes, producing both pro- and anti-inflammatory cytokines and developing macrophages with pro- and anti-inflammatory phenotype[3].

Research into the role of inflammation in cardiovascular disease has found increased monocyte counts in patients with a myocardial infarction and other forms of acute cardiovascular pathology[1, 4, 5]. Monocyte-derived “foam cell” macrophages are a substrate for atherosclerosis and thus facilitate the progress to myocardial infarction. Overall, monocytes have been used as indicators of prognosis in humans with their high numbers being associated with increased risk of recurrent myocardial infarction, hospitalization and cardiac death[1]. Available data indicate that monocyte mobilization in acute cardiac disease does not simply reflect a response to cardiac damage, as they are actively involved in the pathological processes themselves [6, 7].

Introduction of oral anticoagulation has dramatically reduced the risk of stroke. However, the contemporary outcomes in Atrial Fibrillation (AF) are increasingly driven by non-embolic events and complication of oral anticoagulation (bleeding). The role of monocytes in determining outcomes amongst AF patients is unknown. Such data could help identify patients at high risk of adverse outcomes and subsequently highlight those in need of targeted therapy to control cardiovascular risk factors as well as novel therapeutic strategies aimed at modulating the inflammatory response in AF patients.

Our aim was to investigate the prognostic roles of monocyte counts in AF for the occurrence of death, major adverse cardiovascular events (MACE), stroke and systemic embolism (SSE), as well as significant bleeding events in a longer term observational study cohort of AF patients. We tested the hypothesis that high monocyte counts confer an increased risk of these adverse outcome.

## Methods

Patients with documented AF were recruited from outpatient Atrial fibrillation clinics in Sandwell and West Birmingham Hospitals Trust and Oral anticoagulation clinics in the West Birmingham area between August 2008 and August 2010 (Table 1). There has been no patient selection based on co-morbidities. All recruited patients were included into the analysis if they had data on monocyte counts after the diagnosis of AF (36 [4%] of the patients were excluded for this reason). A total of 881 patients with data on monocyte counts were included in this analysis. Data on blood monocyte counts during routine appointments after a diagnosis of AF were obtained from clinical records. Monocyte data from acute admissions were not included. Follow up monocyte data were collected from routine appointments at one-year time or nearest later date and were available for 670 patients.

**Table 1:** Clinical characteristics and study outcomes of patients at baseline and follow up.

	All (n = 881)	OAC (n = 524)	No OAC (n = 357)
Age (years)	71 (62–78)	72 (64–79)*	68 (58–78)
Male sex (n)	524 (59%)	308 (59%)	216 (61%)
Non-White ethnicity (n)	171 (20%)	82 (16%)*	89 (25%)
CHA_2_DS_2_-VASc score	3.3±1.5	3.5±1.4*	3.0±1.6
HAS-BLED score	1.4±0.8	1.2±0.7*	1.7±0.9
Monocytes (per μL)	0.45 (0.36–0.58)	0.36 (0.46–0.58)	0.36 (0.45–0.58)
Monocyte count >800 per μL (n)	69 (7.8%)	34 (6.5%)	35 (9.8%)
Body mass index (kg/m^2^)	28 (25–33)	25 (29–34)	25 (28–32)
Systolic BP (mm Hg)	137 (123–152)	122 (137–151)	124 (138–153)
Diastolic BP (mm Hg)	80 (72–90)	72 (80–90)	71 (80–90)
Creatinine (μmol/L)	90 (76–106)	91 (78–107)	90 (74–105)
Atrial fibrillation type			
Paroxysmal	337 (38%)	137 (26%)*	200 (56%)
Persistent	116 (13%)	84 (16%)	32 (9%)
Long standing persistent or permanent	428 (49%)	303 (58%)	125 (35%)
Past medical history			
Myocardial infarction (n)	129 (14%)	72 (13%)	57 (15%)
Congestive heart failure (n)	153 (17%)	112 (21%)*	41 (11%)
Coronary artery bypass grafting (n)	30 (3%)	19 (4%)	11 (3%)
Percutaneous coronary (n)	30 (3%)	14 (3%)	16 (4%)
Peripheral artery disease (n)	26 (4%)	15 (3%)	11 (4%)
End-stage renal failure (n)	5 (1%)	1 (0.2%)	4 (1%)
End-stage liver failure (n)	3 (0.3%)	1 (0.2%)	2 (1%)
Alcohol excess (n)	32 (4%)	12 (2%)*	20 (6%)
Pharmacotherapy			
Beta-blocker (n)	396 (45%)	234 (45%)	162 (45%)
Amiodarone or flecainide (n)	95 (11%)	47 (9%)*	48 (13%)
Angiotensin receptor blocker (n)	212 (24%)	126 (24%)*	86 (24%)
ACE inhibitors (n)	320 (36%)	209 (40%)	111 (31%)
Anti-anginal agents (n)	73 (8%)	38 (7%)	35 (10%)
Statin (n)	342 (39%)	200 (38%)	142 (40%)
Digoxin (n)	238 (27%)	181 (35%)*	57 (16%)
Dihydropyridine CCA (n)	191 (22%)	107 (20%)	84 (24%)
Non-dihydropyridine CCA (n)	203 (23%)	127 (24%)	76 (21%)
Diuretics	393 (45%)	268 (51%)*	125 (35%)
Outcomes			
Death (n)	390 (44%)	245 (47%)	145 (41%)
MACE (n)	424 (48%)	262 (50%)	162 (45%)
Stroke or systemic embolism (n)	79 (9%)	42 (8%)	37 (10%)
Major bleeding (n)	43 (5%)	28 (5%)	15 (4%)

Age at the time of monocyte assessment, other clinical characteristics at the time of recruitment;

*p<0.05 vs. patients not receiving OAC; ACE, angiotensin converting enzyme; BP, blood pressure; CCA, calcium channel antagonist; MACE, major adverse cardiovascular events; OAC, oral anticoagulation

Patients were prospectively followed up with clinical outcomes recorded based on clinical records. The study outcomes were (i) any-cause death; (ii) major adverse cardiovascular events (MACE: first event of death, myocardial infarction, ischemic stroke, transient ischemic attacks (TIA) or other systemic embolism); (iii) stroke, TIA or other systemic embolism (SSE); and (iv) major bleeding (including hemorrhagic stroke). The study was approved by the Black Country Research and Ethics Committee, UK and the study complied with the Declaration of Helsinki. All patients provided written informed consent for participation in the study.

### Statistical analysis

Descriptive statistics are presented as medians (interquartile range) for continuous variables or numbers (percentage to the total population) for categorical variables. Predictive value of monocyte counts for the study outcomes were assessed using univariate and multivariable Cox regression models with monocyte levels dichotomized as above or below the 4^th^ quartile of their baseline levels (i.e., 580 per μL). Separate analyses were done for predictive value of persistent (i.e. both the baseline and follow up) monocyte counts ≥580 per μL. Multivariable analyses included adjustments for CHA_2_DS_2_-VASc score (congestive heart failure, hypertension [BP consistently>140/90], age≥75, diabetes mellitus, prior stroke or TIA or thromboembolism) values for death, MACE and SSE, and an adjustment for HASBLED score (uncontrolled hypertension [SBP>160mmHg], abnormal renal function [dialysis, transplant, creatinine>2.26mg/dl or >200μmol/L, abnormal liver function [cirrhosis or bilirubin>2x normal or AST/ALP/AP >3x normal], prior history of stroke, prior major bleeding or predisposition to bleeding, liable INR [time in therapeutic range <60%], age>65 years, prior alcohol or drug usage [≥8 drinks/week], medication usage predisposing to bleeding) values for major bleeding events. To establish interactions between the monocyte related outcomes and use of oral anticoagulation, non-White ethnicity, advanced age (i.e. 75 years or older) and presence of long standing persistent or permanent form of AF, we ran additional separate regression models for each of the parameters and its interactions with the monocyte status added to the multivariable models above. P-values of <0.05 were considered as statistically significant. STATA 13 (STATA Inc., USA) software was used for statistical analyses.

## Results

We included 881 patients with a median follow up of 7.2 (4.8–10) years. The index monocyte assessment was done 0.7 (0.1–3.0) years after the initial diagnosis of AF. Patient demographic and clinical characteristics are presented in Table 1. At the time of the recruitment 525 (59%) patients received OAC. Follow up monocyte data were available for 670 patients with 23 (17–33) months duration from the first sample. Persistent monocyte count ≥580 per μL was recorded in 84 (13%) patients.

### All-cause death

**Fig 1:**
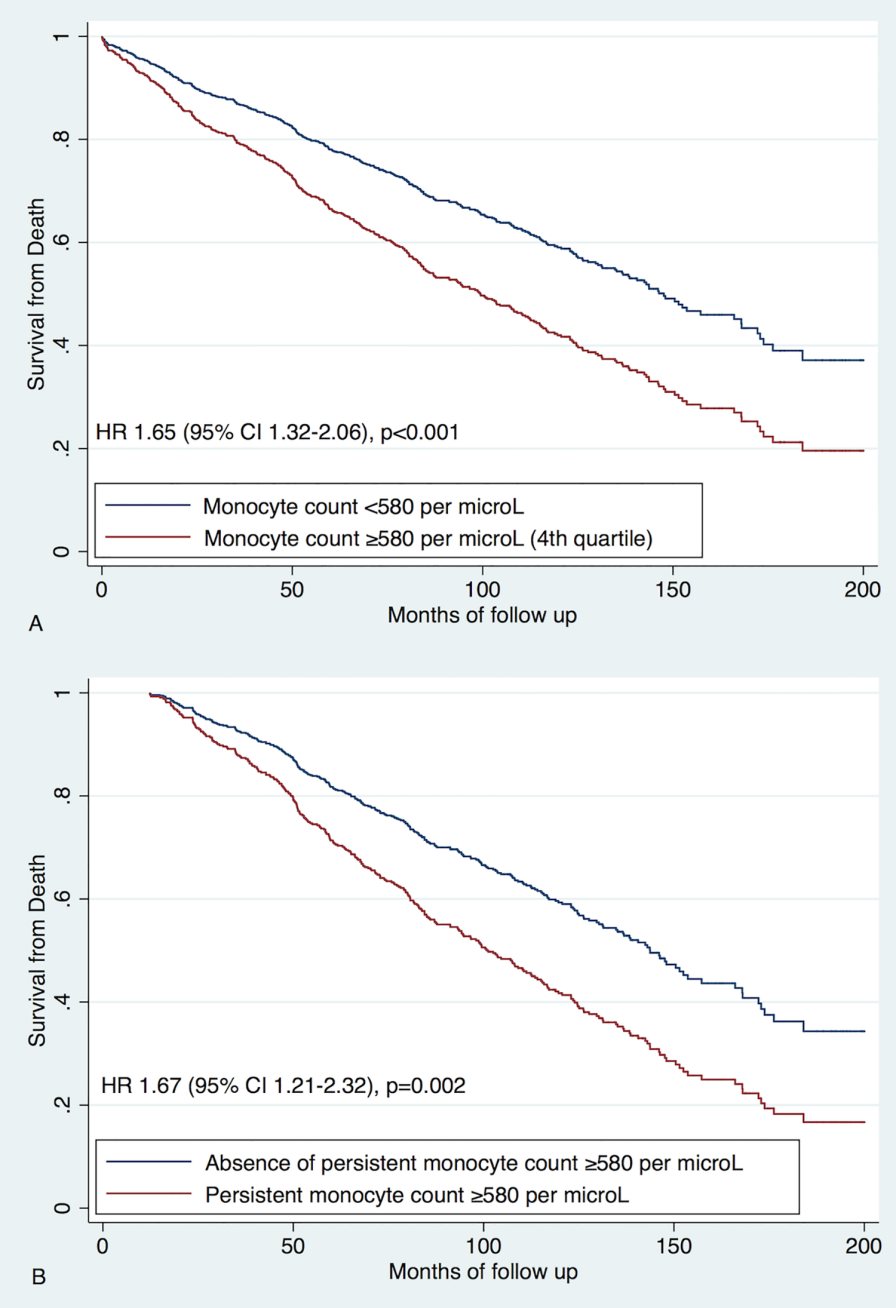
Predictive value of monocytes for all-cause death. A. Univariate analysis of baseline monocyte levels; Analysis of baseline monocytes adjusted for CHA_2_DS_2_-VASc score; B. Univariate analysis of follow up monocyte data; Analysis of follow up monocytes data adjusted for CHA_2_DS_2_-VASc score.

Three hundred and ninety (44%) patients died during follow up (Table 1). The 4^th^ monocyte quartile (i.e., ≥580 per μL, vs. other quartiles) was associated with increased risk of death on univariate analysis (hazard ratio [HR] 1.65, 95% confidence interval [CI] 1.32–2.06, p<0.001) and after adjustment for CHA_2_DS_2_-VASc score (HR 1.64, 95% CI 1.31–2.05, p<0.001) (Fig 1). Persistent monocyte levels ≥580 per μL were associated with further increase of risk of death (HR 1.67, 95% CI 1.21–2.32, p = 0.002 for univariate analysis and HR 1.52, 95% CI 1.10–2.11, p = 0.011 after adjustment for CHA_2_DS_2_-VASc score).

### Major adverse cardiovascular events

**Fig 2:**
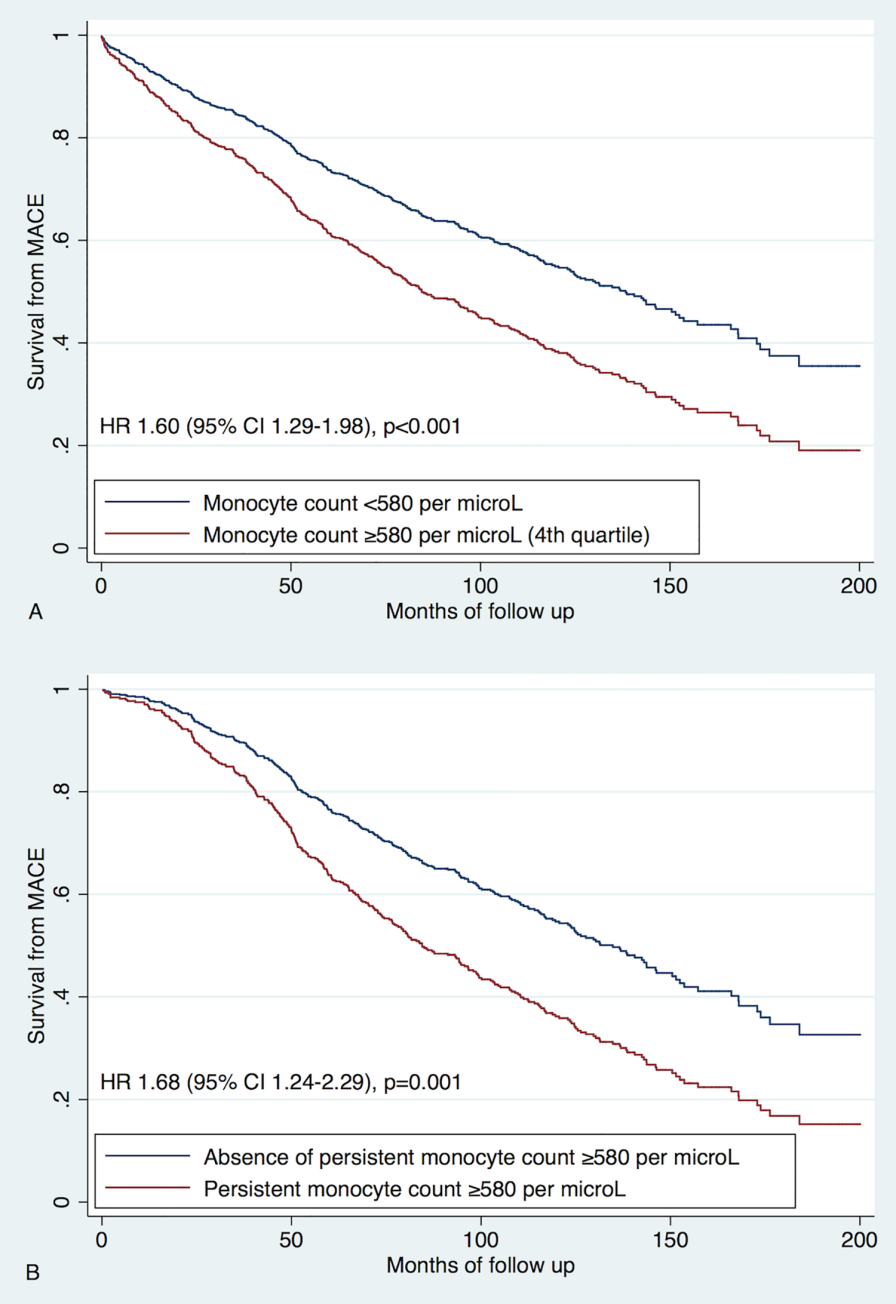
Predictive value of monocytes for major adverse cardiovascular events. A. Univariate analysis of baseline monocyte levels; Analysis of baseline monocytes adjusted for CHA_2_DS_2_-VASc score; B. Univariate analysis of follow up monocyte data; Analysis of follow up monocytes data adjusted for CHA_2_DS_2_-VASc score.

MACE occurred in 424 (48%) patients (Table 1). The highest monocyte quartile was associated with an increased risk of MACE on univariate analysis (HR 1.60, 95% CI 1.29–1.98, p<0.001) and after adjustment for CHA_2_DS_2_-VASc score (HR 1.58, 95% CI 1.28–1.96, p<0.001) (Fig 2). Persistent monocyte levels ≥580 per μL were associated with further increase of risk of death (HR 1.68, 95% CI 1.24–2.29, p = 0.001 for univariate analysis and HR 1.54, 95% CI 1.13–2.09, p = 0.006 after adjustment for CHA_2_DS_2_-VASc score).

### Stroke and systemic embolism

**Fig 3:**
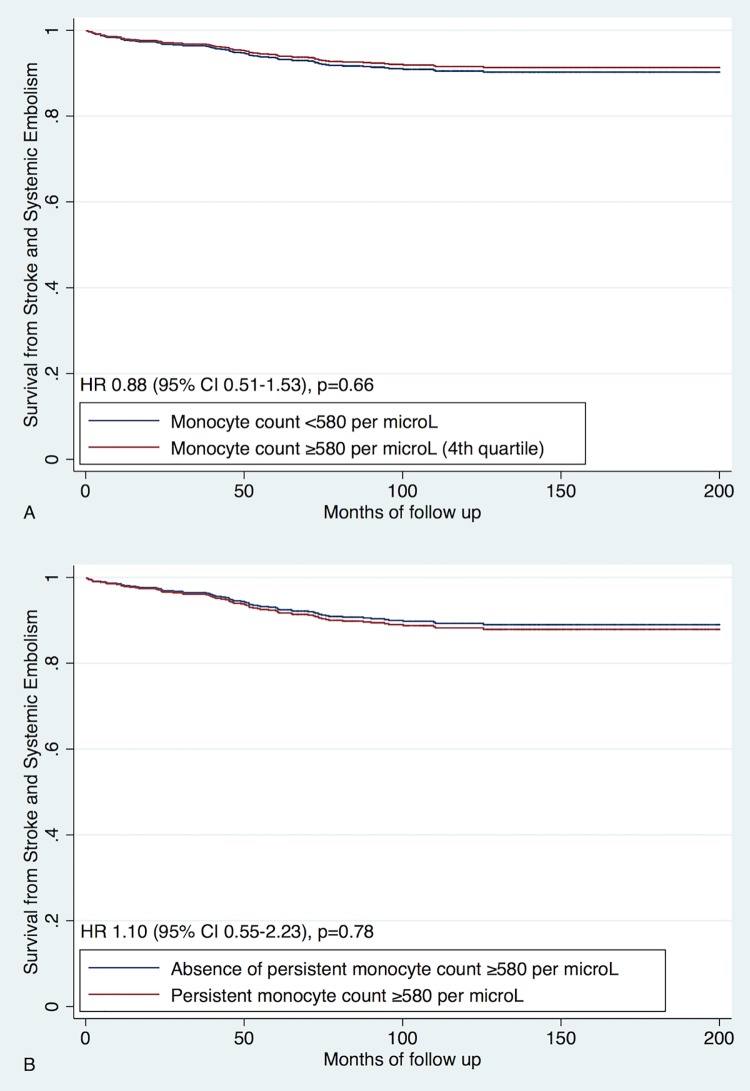
Predictive value of monocytes for stroke and systemic embolism. A. Univariate analysis of baseline monocyte levels; Analysis of baseline monocytes adjusted for CHA_2_DS_2_-VASc score; B. Univariate analysis of follow up monocyte data; Analysis of follow up monocytes data adjusted for CHA_2_DS_2_-VASc score.

SSE occurred in 79 (9%) patients (Table 1). Monocyte counts were not predictive of SSE (for baseline values: univariate analysis HR 0.88, 95% CI 0.51–1.53, p = 0.66, multivariate analysis HR 0.85, 95% CI 0.49–1.48, p = 0.57; for persistent monocyte levels ≥580 per μL: univariate analysis HR 1.10, 95% CI 0.55–2.23, p = 0.78, multivariate analysis HR 1.03, 95% CI 0.51–2.08, p>0.93) (Fig 3).

### Major bleeding (including hemorrhagic stroke)

**Fig 4:**
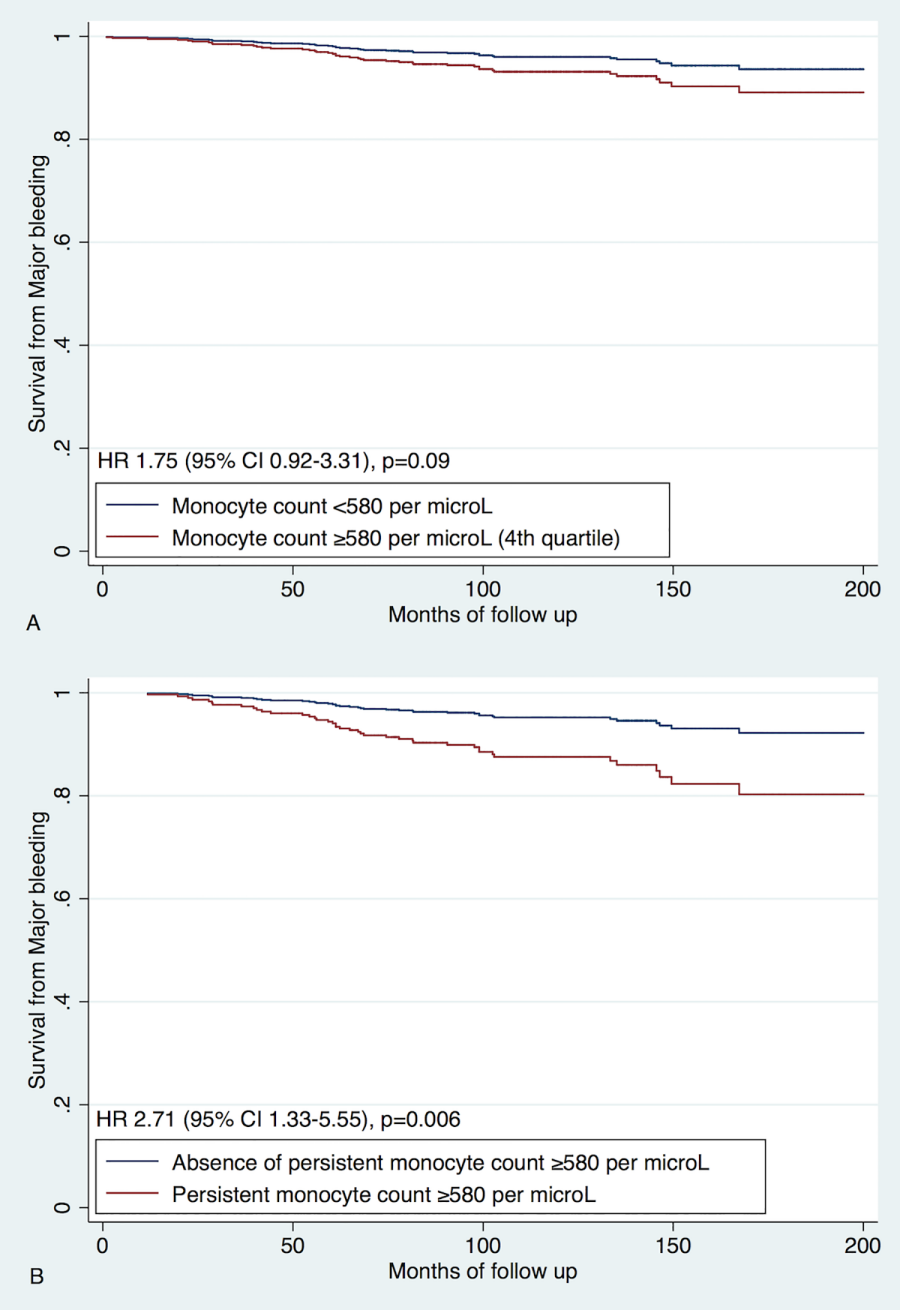
Predictive value of monocytes for major bleeding. A. Univariate analysis of baseline monocyte levels; Analysis of baseline monocytes adjusted for CHA_2_DS_2_-VASc score; B. Univariate analysis of follow up monocyte data; Analysis of follow up monocytes data adjusted for CHA_2_DS_2_-VASc score.

Major bleeding events occurred in 43 (5%) patients (Table 1). The highest baseline monocyte quartile showed a non-significant trend towards the higher risk of major bleeding (HR 1.75, 95% CI 0.92–3.31, p = 0.09 for univariate and HR 1.75, 95% CI 0.93–3.32, p = 0.09 for multivariate analysis) (Fig 4). Persistent monocyte levels ≥580 per μL were associated with a significant increase in major bleeding events (HR 2.71, 95% CI 1.33–5.55, p = 0.006 for univariate analysis, and HR 2.77, 95% CI 1.36–5.67, p = 0.005 after adjustment for HAS-BLED score).

### Interactions of monocyte status with age, ethnicity and AF characteristics

On additional regression models, there were no significant interactions between the monocyte status and use of oral anticoagulants, non-White ethnicity, advanced age and presence of long standing persistent or permanent AF for all tested outcomes (Table 2).

**Table 2:** Additional predictors of outcome and their interactions between monocytes.

	Baseline			Follow up		
	HR (95% CI)	p for variable	p for interaction	HR (95% CI)	p for variable	p for interaction
*Death*						
OAC use	0.77 (0.60–0.99)	0.040	0.93	0.62 (0.48–0.81)	<0.001	0.50
Non-White	1.54 (1.12–2.13)	0.009	0.26	1.32 (0.96–1.83)	0.09	0.46
Age ≥75 years	2.20 (1.65–2.91)	<0.001	0.89	2.63 (1.93–3.60)	<0.001	0.56
Long standing persistent or permanent AF	1.40 (1.06–1.83)	0.016	0.15	1.56 (1.15–2.10)	0.004	0.92
Major adverse cardiovascular events						
OAC use	0.78 (0.62–0.99)	0.044	0.82	0.65 (0.50–0.83)	0.001	0.68
Non-White	1.40 (1.03–1.89)	0.030	0.12	1.20 (0.89–1.63)	0.24	0.09
Age ≥75 years	1.78 (1.36–2.32)	<0.001	0.97	2.05 (1.54–2.74)	0.000	0.28
Long standing persistent or permanent AF	1.33 (1.03–1.72)	0.030	0.17	1.47 (1.11–1.94)	0.008	0.91
Stroke or systemic embolism						
OAC use	0.88 (0.52–1.48)	0.63	0.29	0.79 (0.47–1.32)	0.36	0.33
Non-White	0.80 (0.45–1.42)	0.45	0.80	0.85 (0.48–1.52)	0.58	0.39
Age ≥75 years	0.59 (0.34–1.02)	0.06	0.66	0.63 (0.36–1.09)	0.10	0.98
Long standing persistent or permanent AF	1.07 (0.62–1.86)	0.81	0.19	1.01 (0.58–1.77)	0.97	0.07
Major bleeding						
OAC use	1.11 (0.50–2.46)	0.81	0.81	1.51 (0.66–3.43)	0.33	0.17
Non-White	0.63 (0.28–1.42)	0.26	0.26	0.77 (0.34–1.71)	0.52	0.81
Age ≥75 years	2.07 (0.92–4.67)	0.08	0.55	2.38 (1.04–5.45)	0.04	0.27
Long standing persistent or permanent AF	1.52 (0.67–3.44)	0.32	0.49	1.77 (0.76–4.11)	0.19	0.91

## Translational perspectives

The precise factors driving risk of death and MACE in AF patients with higher (but mostly still within normal limit) monocyte counts are unclear. The likely mechanisms include the inflammatory and profibrotic properties of monocytes. Admittedly the mechanistic links between monocytes and unfavorable outcomes could not be established in this analysis and monocytes could be bystanders of ongoing pathological processes rather than direct cause of the outcome. It is likely contributed by the fact that monocytes are a major source of inflammatory cytokines and reactive oxygen species in the circulation.[8]

With regards to bleeding, fibrinolysis is tightly controlled by a series of cofactors, inhibitors, and receptors.[9] Plasmin is the primary fibrinolytic enzyme, and is activated from plasminogen by either of two primary serine proteases, tissue-type plasminogen activator and urokinase-type plasminogen activator. Urokinase-type plasminogen activator is primarily produced by monocytes and macrophages.[10, 11] Excessive elevation in monocyte counts or their abnormal functional state may shift the tightly controlled local hemostasis state towards the bleeding state. Monocyte capacity to produce matrix metalloproteases may further amplify the process and thus overcome procoagulant monocyte properties.[12–14]

Physicians in clinical practice should be aware that higher monocyte counts signal high risk of major bleeding in the AF population. This can aid in the decision-making process when considering oral anticoagulation and measures to reduce bleeding risk. Future areas of research into this field should aim to delineate possible roles of monocyte subsets in bleeding risk and possible therapeutic measures to attenuate this bleeding risk.

## Conclusion

High monocyte counts independently predict the occurrence of MACE, major bleeding and mortality, but not SSE. This study demonstrates for the first time a strong association between monocyte counts and the subsequent adverse outcomes of all cause death, MACE and major bleeding in patients with AF. The similar rates of stroke/SSE in patients on or without oral anticoagulation may be reflective of the younger average age of the population not receiving the treatment. Furthermore, the lower CHA_2_DS_2_-VASc score in this group reflects a lower overall baseline stroke risk. The powerful associations were present despite adjustment for well-established scores for prognostication of thromboembolic and bleeding complications in AF. Indeed, the top quartile of monocyte counts were associated with a 64% rise in all-cause death and 58% increase in risk of MACE even after adjustment for the CHA_2_DS_2_-VASc score, which accounts for major prognosticators of unfavorable cardiovascular outcomes and age. Specifically, persistent monocyte levels at 580 per μL or above were associated with over 50% increase in risk of death or MACE irrespectively of the baseline monocyte status.

The study also demonstrates for the first time that persistently high monocyte levels (i.e., ≥580 per μL) are associated with almost 3-fold increase in major bleeding events. These data support clinical relevance of previous, mostly experimental data demonstrating pro-fibrinolytic activity of monocytes.[12] Of note, risk of stroke was not related to monocyte counts, despite the increased rates of other cardiovascular events. This may reflect the multidirectional effects of monocytes, including excessive inflammation, driving risk of myocardial infarction and death, and exaggerated fibrinolysis and connective tissue degradation predisposing to bleeding. Accumulating data point toward the potential implication monocyte in bleeding complications. This can be largely mediated by their three functions: production of matrix metalloproteases, that can break the barrier between the vascular lumen and the surrounding space, (ii) clot resolution via phagocytosis and (iii) providing surface for fibrinolysis. Further research is needed to get further insights into these processes.[12]

Understanding the pathophysiological mechanisms involved would help understand the relationships between monocytes, and adverse thrombotic and bleeding outcomes in AF patients.

### Limitations

The study has several limitations. Although confounding factors were considered with regards to AF-related risk stratification, possible effects of various co-morbidities (overt or subclinical) affecting monocyte counts have not been analysed. This is an observational analysis, and mechanistic insights into molecular mechanisms of the findings and monocyte functional characteristics have not been studied.[15] As the study included patients with monocytes measured as part of routine clinical management, this could lead to some selection bias due to not inclusion of patients with no full blood count tested at all during the routine assessment. The medications used by the participants are likely undergone modifications during the years of followed and all changes may not have been captured by this study.

## Abbreviations

AF: atrial fibrillation
MACE: major adverse clinical events
OAC: oral anticoagulation
SSE: stroke and systemic embolism
TIA: transient ischemic attack
